# Supplementary material for: Seesaw conformations of Npl4 in the human p97 complex and the inhibitory mechanism of a disulfiram derivative
Source: Nat Commun. 2021 Jan 5;12:121. doi: 10.1038/s41467-020-20359-x (PMC7785736; doi:10.1038/s41467-020-20359-x)
Supplement: Supplementary file 1 — Supplementary Information [file 41467_2020_20359_MOESM1_ESM.pdf]

Supplementary Information

Seesaw Conformations of Npl4 in the Human p97 Complex and the Inhibitory Mechanism of a Disulfiram Derivative

Man Pan[1,4], Qingyun Zheng[2,4], Yuanyuan Yu[1,4], Huasong Ai[2], Yuan Xie[1], Xin Zeng[3], Chu Wang[3], Lei Liu[2]*, Minglei Zhao[1]*

[1] Department of Biochemistry and Molecular Biology, The University of Chicago, Chicago, IL 60637, USA.

[2] Tsinghua-Peking Center for Life Sciences, Department of Chemistry, Tsinghua University, Beijing 100084, China.

[3] Peking-Tsinghua Center for Life Sciences, College of Chemistry and Molecular Engineering, Peking University, Beijing 100871, China

[4] These authors contributed equally to the work.

* To whom correspondence should be addressed: mlzhao@uchicago.edu (M. Z.), lliu@mail.tsinghua.edu.cn (L. L.)

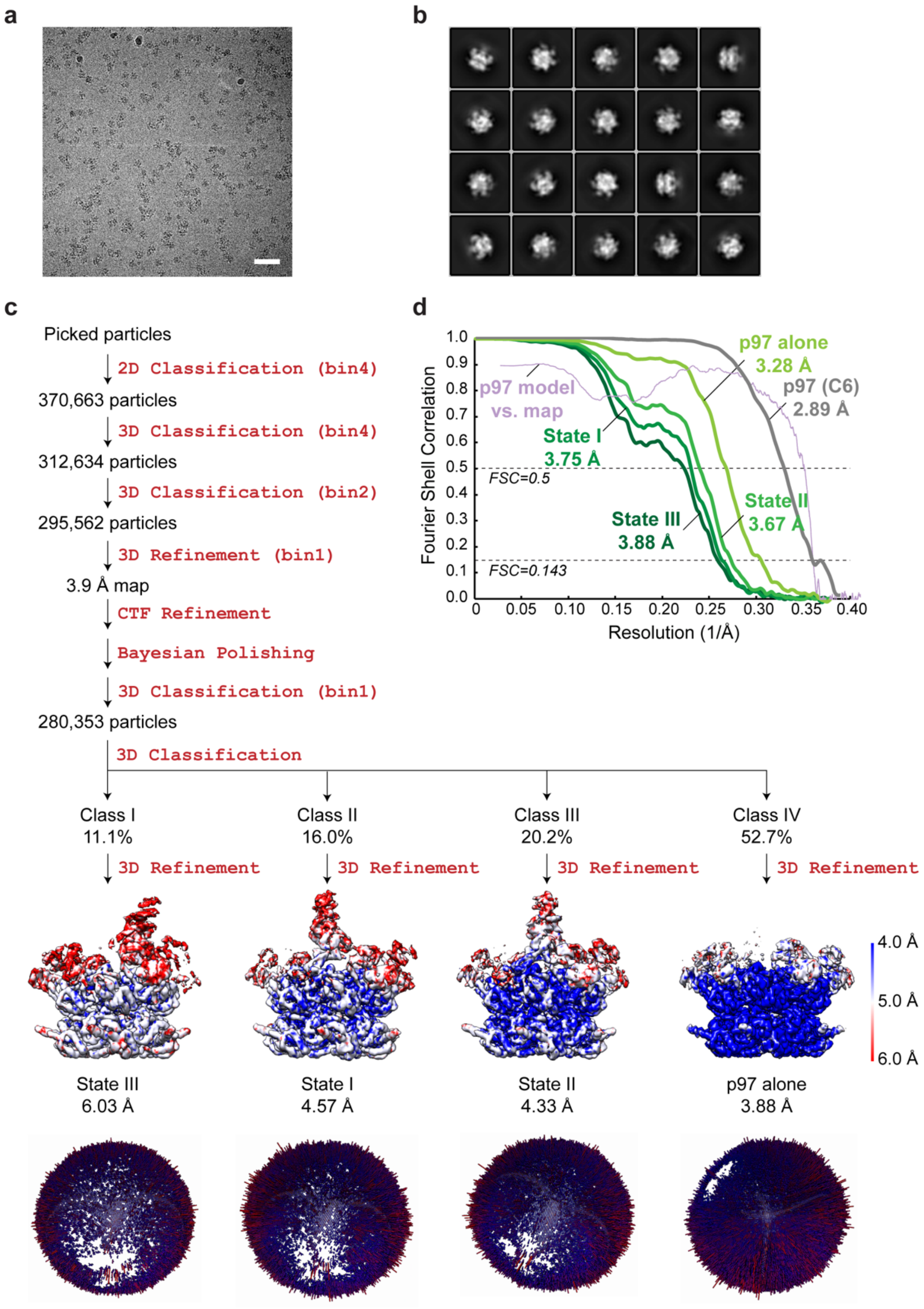

a
b
c
Picked particles
2D Classification (bin4)
370,663 particles
3D Classification (bin4)
312,634 particles
3D Classification (bin2)
295,562 particles
3D Refinement (bin1)
3.9 Å map
CTF Refinement
Bayesian Polishing
3D Classification (bin1)
280,353 particles
3D Classification
Class I
11.1%
Class II
16.0%
Class III
20.2%
Class IV
52.7%
3D Refinement
State III
6.03 Å
State I
4.57 Å
State II
4.33 Å
p97 alone
3.88 Å
4.0 Å
5.0 Å
6.0 Å
d
Fourier Shell Correlation
Resolution (1/Å)
p97 alone
3.28 Å
p97 (C6)
2.89 Å
p97 model
vs. map
State I
3.75 Å
State II
3.67 Å
State III
3.88 Å
FSC=0.5
FSC=0.143

Supplementary Figure 1: Single-particle cryo-EM analyses for the complex of p97 and Npl4/Ufd1.

(**a**) A representative micrograph. The scale bar corresponds to 50 nm. (**b**) Representative 2D class averages. The box edge corresponds to 340 Å. (**c**) The workflow of data processing. The dataset was subjected to particle selection, 2D classification, and multiple rounds of 3D classification. Four maps including three different states of the complex and p97 alone were resolved. The maps were shown in similar orientation aligned in UCSF Chimera[1]. The local resolutions of the maps were calculated using LocalRes[2]. Resolutions after Relion 3D refinement and before post processing were listed. The distributions of the Euler angle (half sphere) corresponding to the local resolution maps were shown. (**d**) Fourier shell correlation (FSC) curves of the masked maps after Relion post processing. The resolutions were determined by the FSC=0.143 criterion. The model (p97 structure deposited) vs. map (masked around p97 and C6 averaged) FSC curve was also shown.

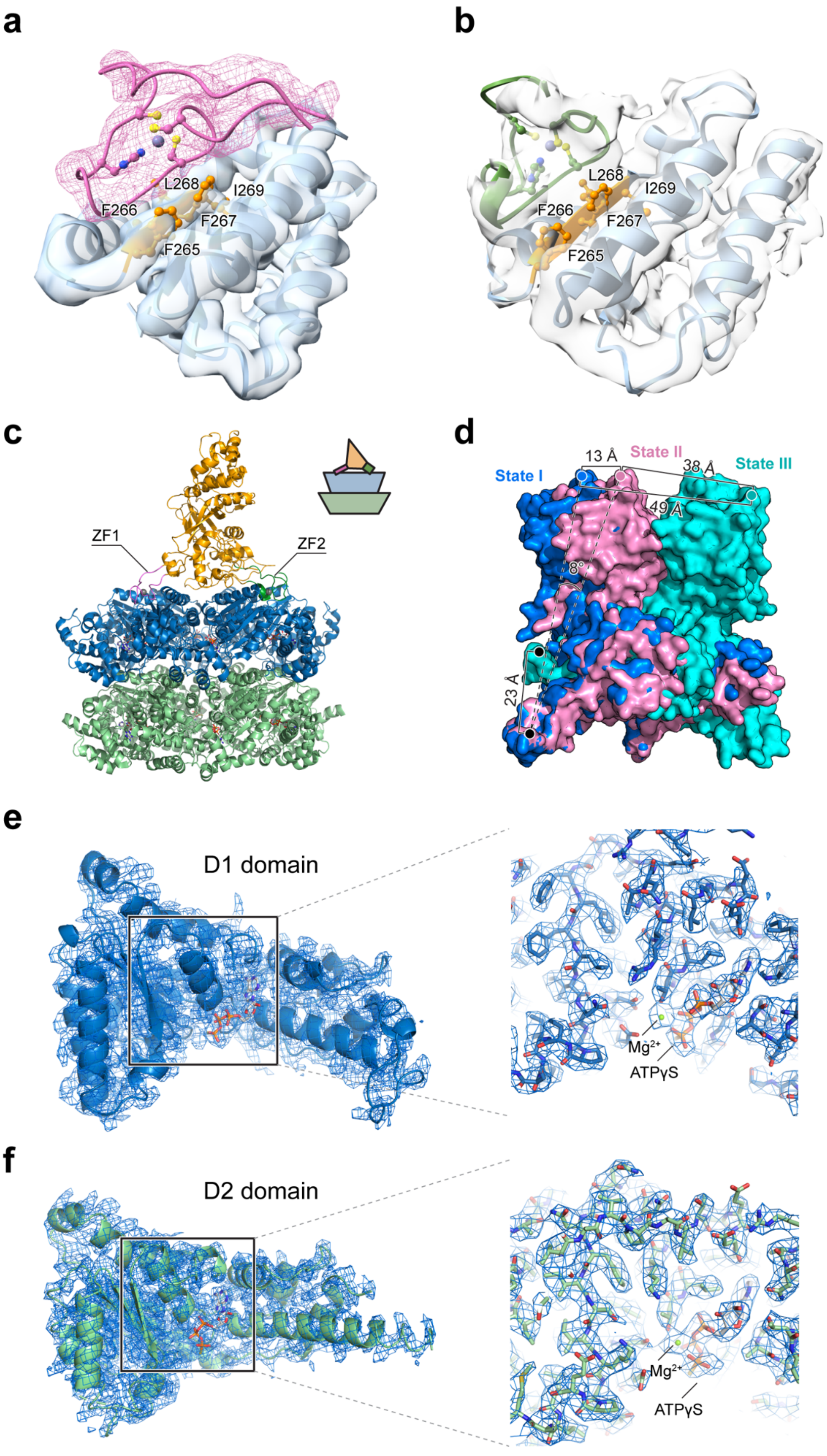
a
L268
I269
F266
F267
F265
b
L268
I269
F266
F267
F265
c
ZF1
ZF2
d
13 Å
State II
38 Å
State III
State I
49 Å
8°
23 Å
e
D1 domain
Mg2+
ATPγS
f
D2 domain
Mg2+
ATPγS

Supplementary Figure 2: Interactions between the zinc finger motifs of Npl4 and p97.

(**a**) Interactions between ZF1 and the D1 domain of p97 (from State II of the p97-Npl4/Ufd1 complex). ZF1 forms a β-sheet with the large subunit of the D1 domain. The contacting β-strand in the D1 domain is colored in orange with the five hydrophobic residues (FFFLI) labeled. The map quality is good enough to enable model building of ZF1. (**b**) Interactions between ZF2 and the D1 domain of p97 (from State III of the p97-Npl4/Ufd1 complex). ZF2 also forms a β-sheet with the large subunit of the D1 domain at the same location. The map quality is not sufficient to allow model building of ZF2, therefore a homology model was docked into the density as a rigid body. (**c**) Structure of Cdc48-Npl4 complex from thermophilic fungi (PDB code: 6CHS). The position of Npl4 relative to Cdc48 is illustrated in the cartoon. Note that both zinc finger motifs are interacting with the D1 ring of Cdc48. (**d**) Measurements of conformational changes of Npl4 from the three states. The structures were aligned based on the D1 and D2 rings of p97. (**e**) A zoom view of the D1 domain showing the density around ATPγS (C6 averaged map masked around p97, 5.0 rmsd). (**f**) A zoom view of the D2 domain showing the density around ATPγS (C6 averaged map masked around p97, 5.0 rmsd). Same color code is used as in Fig. 1.

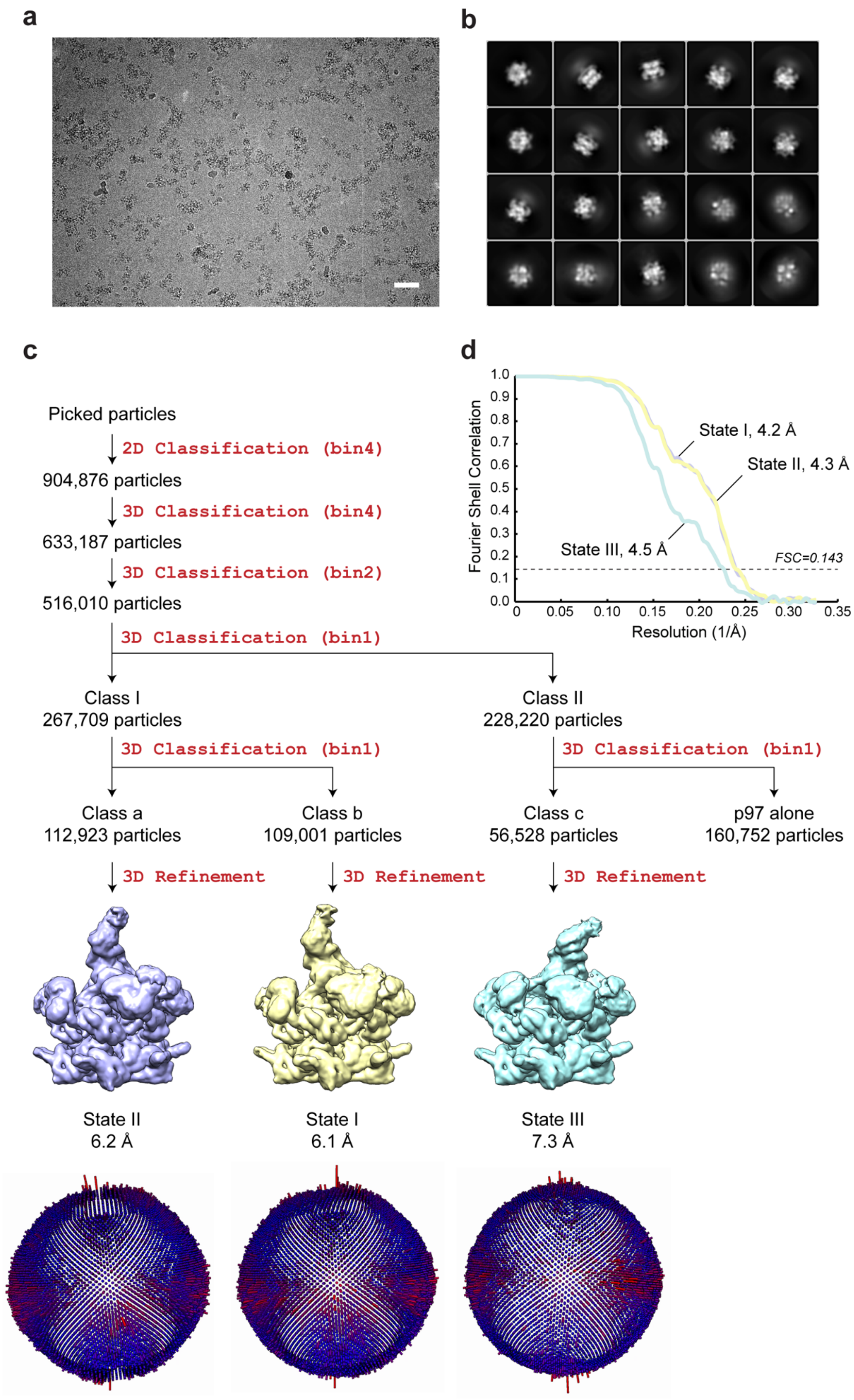
a
b
c
d
Picked particles
2D Classification (bin4)
904,876 particles
3D Classification (bin4)
633,187 particles
3D Classification (bin2)
516,010 particles
3D Classification (bin1)
Class I
267,709 particles
Class II
228,220 particles
3D Classification (bin1)
3D Classification (bin1)
Class a
112,923 particles
Class b
109,001 particles
Class c
56,528 particles
p97 alone
160,752 particles
3D Refinement
3D Refinement
3D Refinement
State II
6.2 Å
State I
6.1 Å
State III
7.3 Å
Fourier Shell Correlation
Resolution (1/Å)
State I, 4.2 Å
State II, 4.3 Å
State III, 4.5 Å
FSC=0.143
1.0
0.9
0.8
0.7
0.6
0.5
0.4
0.3
0.2
0.1
0.0
0
0.05
0.10
0.15
0.20
0.25
0.30
0.35

Supplementary Figure 3: Single-particle cryo-EM analyses for the complex of p97, Npl4/Ufd1, and ubiquitinated Ub-Eos.

(**a**) A representative micrograph. The scale bar corresponds to 50 nm. (**b**) Representative 2D class averages. The box edge corresponds to 386 Å. (**c**) The workflow of data processing. The dataset was subjected to particle selection, 2D classification, and multiple rounds of 3D classification. Three different states of the complex similar to the complex of p97 and Npl4/Ufd1 were resolved. The maps before Relion Post Processing were shown in similar orientation aligned in UCSF Chimera[1]. Resolutions after Relion 3D refinement and before post processing were listed. The distributions of the Euler angle (half sphere) corresponding to the local resolution maps were shown. (**d**) Fourier shell correlation curves of the masked maps after Relion post processing. The resolutions were determined by the FSC=0.143 criterion.

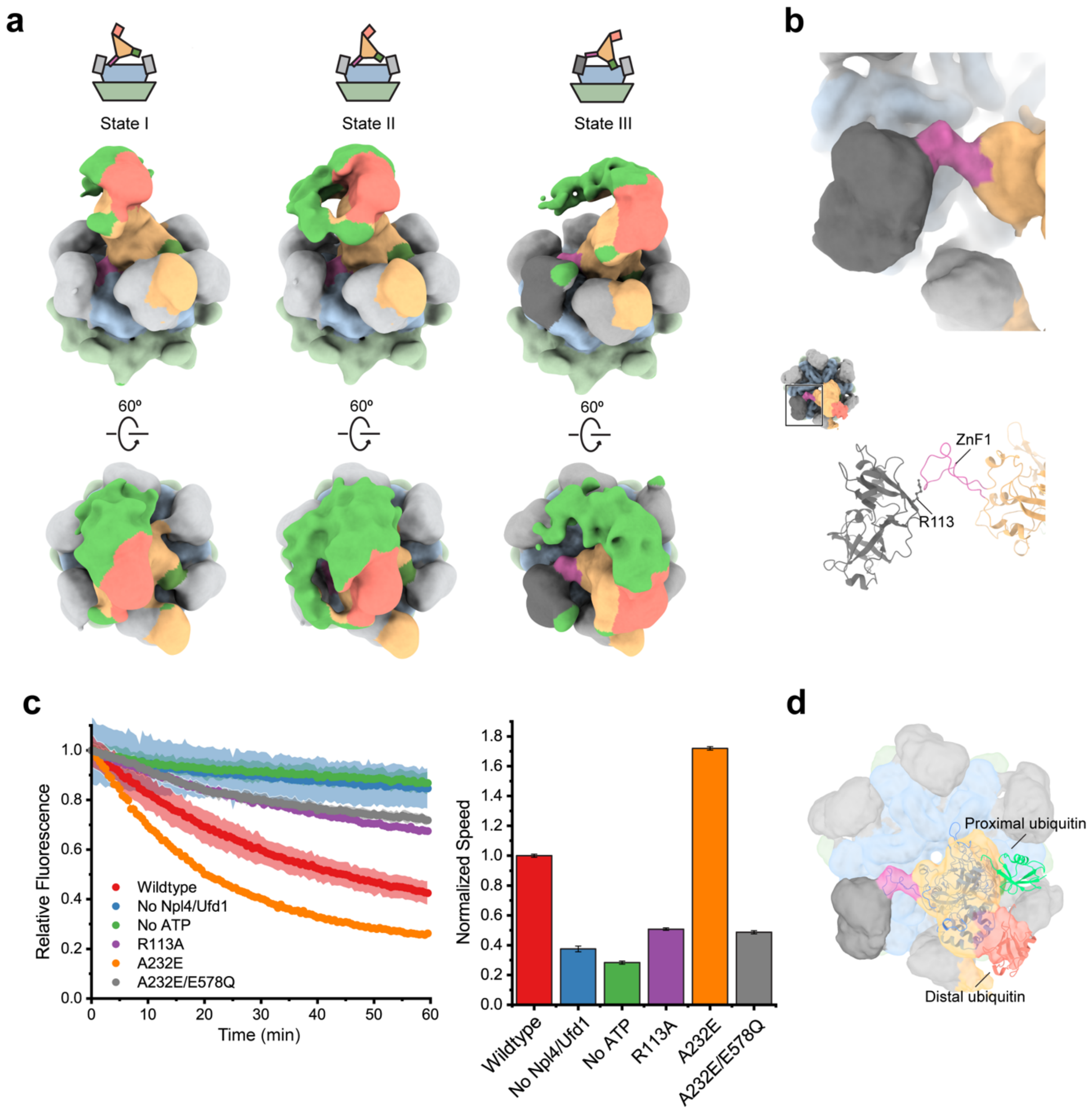

Supplementary Figure 4: Single-particle cryo-EM structure of the complex of p97, Npl4/Ufd1, and ubiquitinated UbEos.

(**a**) Low-pass filtered (15 Å) maps correspond to those in Fig. 2 shown at a lower threshold (0.0035 rmsd). Extra density (bright green) represents the polyubiquitinated Ub-Eos and Ufd1 but could not be resolved due to the extreme flexibility. (**b**) The interactions between ZF1 and the N domain of p97 (from State III of the p97-Npl4/Ufd1-UbEos complex). The resolution is not sufficient to allow the fitting of side chains, therefore the crystal structure of the N domain and the ZF1 derived from panel a was docked into the density as rigid bodies. A tentative interacting residue R113 from the N domain was later mutated to test the relevance of the interaction. (**c**)

The unfolding activity and initial velocity of wild-type p97 and the mutants. The curves are presented as mean values +/- SD from triplicate experiments. The error bars of the columns represent the SEM of the linear fitting. (**d**) A docking of Npl4-Ub subcomplex (from PDB accession code: 6OA9) into State III of p97-Npl4/Ufd1-UbEos map.

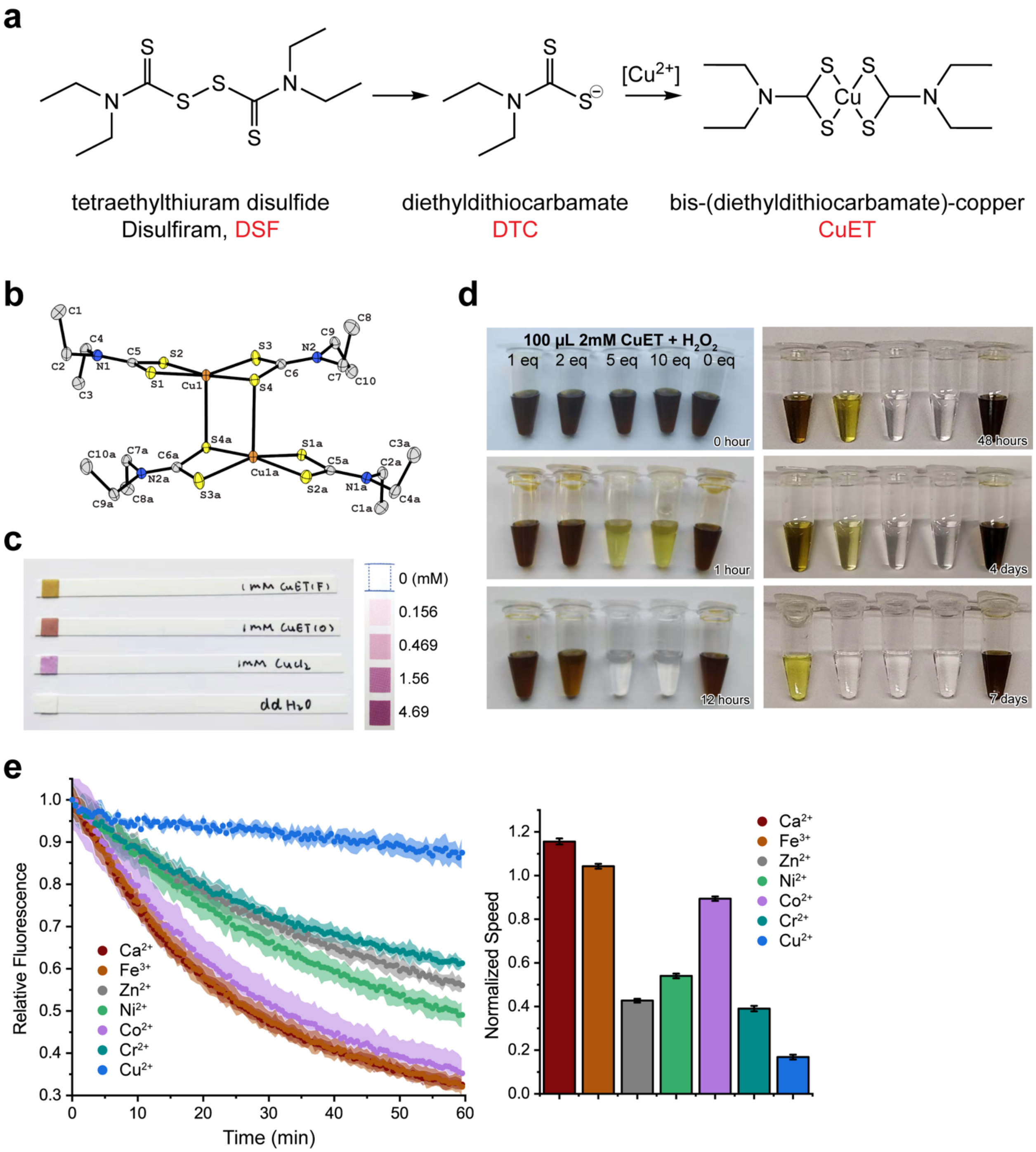

Supplementary Figure 5: Disulfiram derivative CuET releases cupric ion in oxidative condition.

(**a**) Anti-alcohol abuse drug disulfiriram (tetraethylthiuram disulfide, DSF) is metabolized to bis-(diethyldithiocarbamate)-copper (CuET) in the liver[3]. (**b**) The crystal structure of CuET synthesized for this study. (**c**) Fresh CuET (F) solution in DMSO is brown colored. Old CuET (O)

solution (> 7 days at 4 ºC) releases cupric ions as shown by the copper strip with purple tint (Quantofix, Germany). (**d**) CuET solution turns clear with additional hydrogen peroxide that accelerates the copper release in a concentration and time dependent manner. (**e**) The unfolding activity and initial velocity of p97 in the presence of various metal ions at 25 μM. The curves are presented as mean values +/- SD from triplicate experiments. The error bars of the columns represent the SEM of the linear fitting.

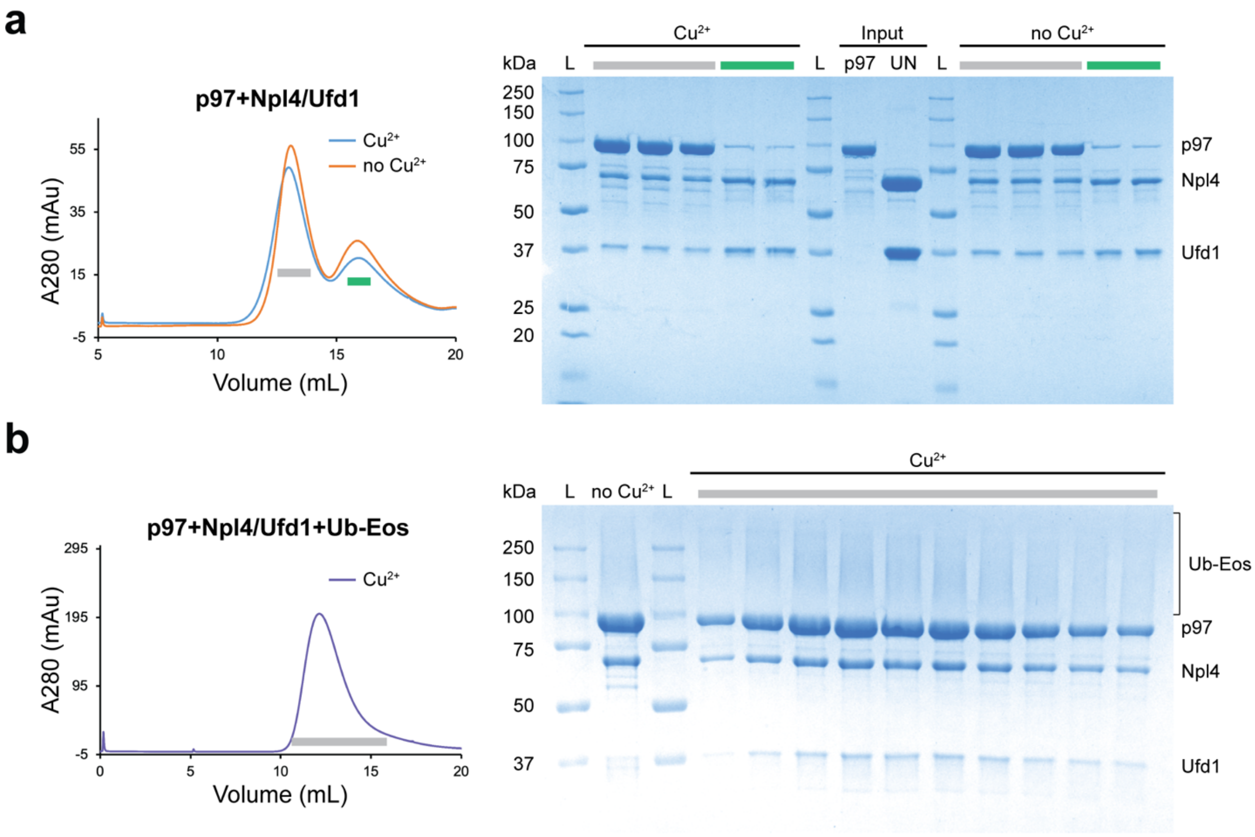

Supplementary Figure 6: Cupric ions do not affect the complex formation of p97, Npl4/Ufd1, and polyubiquitinated Ub-Eos.

(**a**) The size exclusion chromatography profiles and SDS-PAGE gel after incubating p97, Npl4/Ufd1 in the presence (molar ratio 1:3:30) or absence of cupric chloride on ice for one hour. (**b**) The size exclusion chromatography profile and SDS-PAGE gel after incubating preformed p97-Npl4/Ufd1 complex and polyubiquitinated UbEos in the absence and presence of cupric chloride (molar ratio 1:2:10) on ice for one hour.

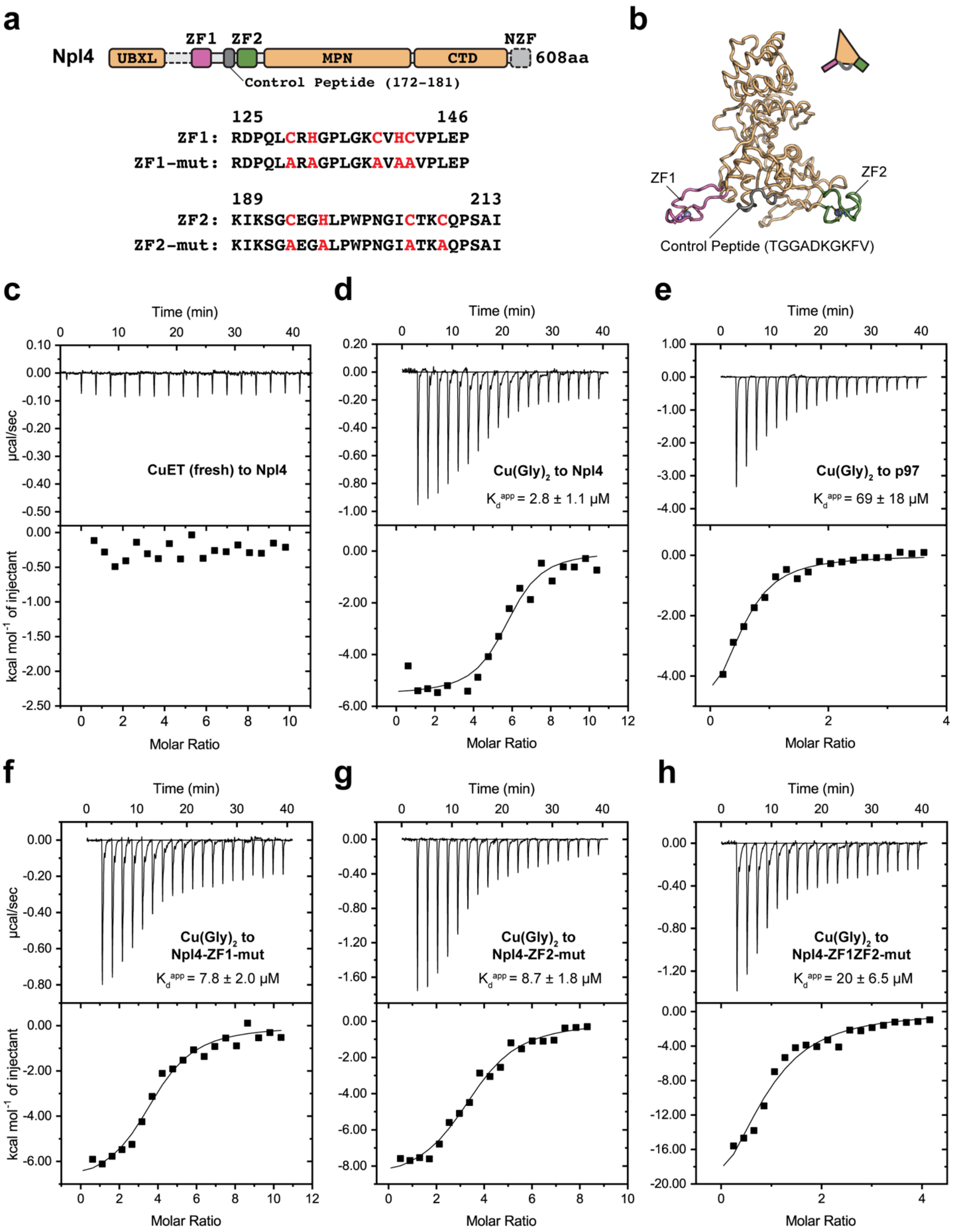
a
Npl4
UBXL
ZF1
ZF2
MPN
CTD
NZF
608aa
Control Peptide (172-181)
125
146
ZF1: RDPQLCRHGPLGKCVHCVPLEP
ZF1-mut: RDPQLARAGPLGKAVAAVPLEP
189
213
ZF2: KIKSGCEGHLPWPNGICTKCQPSAI
ZF2-mut: KIKSGAEGALPWPNGIATKAQPSAI
b
ZF1
ZF2
Control Peptide (TGGADKGKFV)
c
CuET (fresh) to Npl4
d
Cu(Gly)2 to Npl4
Kdapp = 2.8 ± 1.1 μM
e
Cu(Gly)2 to p97
Kdapp = 69 ± 18 μM
f
Cu(Gly)2 to Npl4-ZF1-mut
Kdapp = 7.8 ± 2.0 μM
g
Cu(Gly)2 to Npl4-ZF2-mut
Kdapp = 8.7 ± 1.8 μM
h
Cu(Gly)2 to Npl4-ZF1ZF2-mut
Kdapp = 20 ± 6.5 μM
Time (min)
μcal/sec
kcal mol-1 of injectant
Molar Ratio

Supplementary Figure 7: Cupric ions interact with the zinc finger motifs of Npl4.

(**a**) The zinc finger motif mutants used in this study. (**b**) A homology model of human Npl4 derived from thermophilic fungi homologue (PDB code: 6CDD) using SWISS-MODEL[4]. Three peptides, ZF1 (magenta), ZF2 (green), and control peptide (grey, residues 172-181) were synthesized for the unfolding assay in Fig. 4. (**c-h**) Isothermal titration calorimetry experiments. (**c**) 1 mM CuET (fresh) titrated to 20 μM Npl4; (**d**) 1 mM $Cu(Gly)_2$ titrated to 20 μM Npl4; (**e**) 5 mM $Cu(Gly)_2$ titrated to 29 μM p97; (**f**) 1 mM $Cu(Gly)_2$ titrated to 20 μM Npl4-ZF1-mut; (**g**) 1mM $Cu(Gly)_2$ titrated to 25 μM Npl4-ZF2-mut; (**h**) 0.8 mM $Cu(Gly)_2$ titrated to 40 μM Npl4-ZF1ZF2-mut. Note that only apparent dissociation constant Kd^{app} are given as chemical reactions were taking place during the titration.

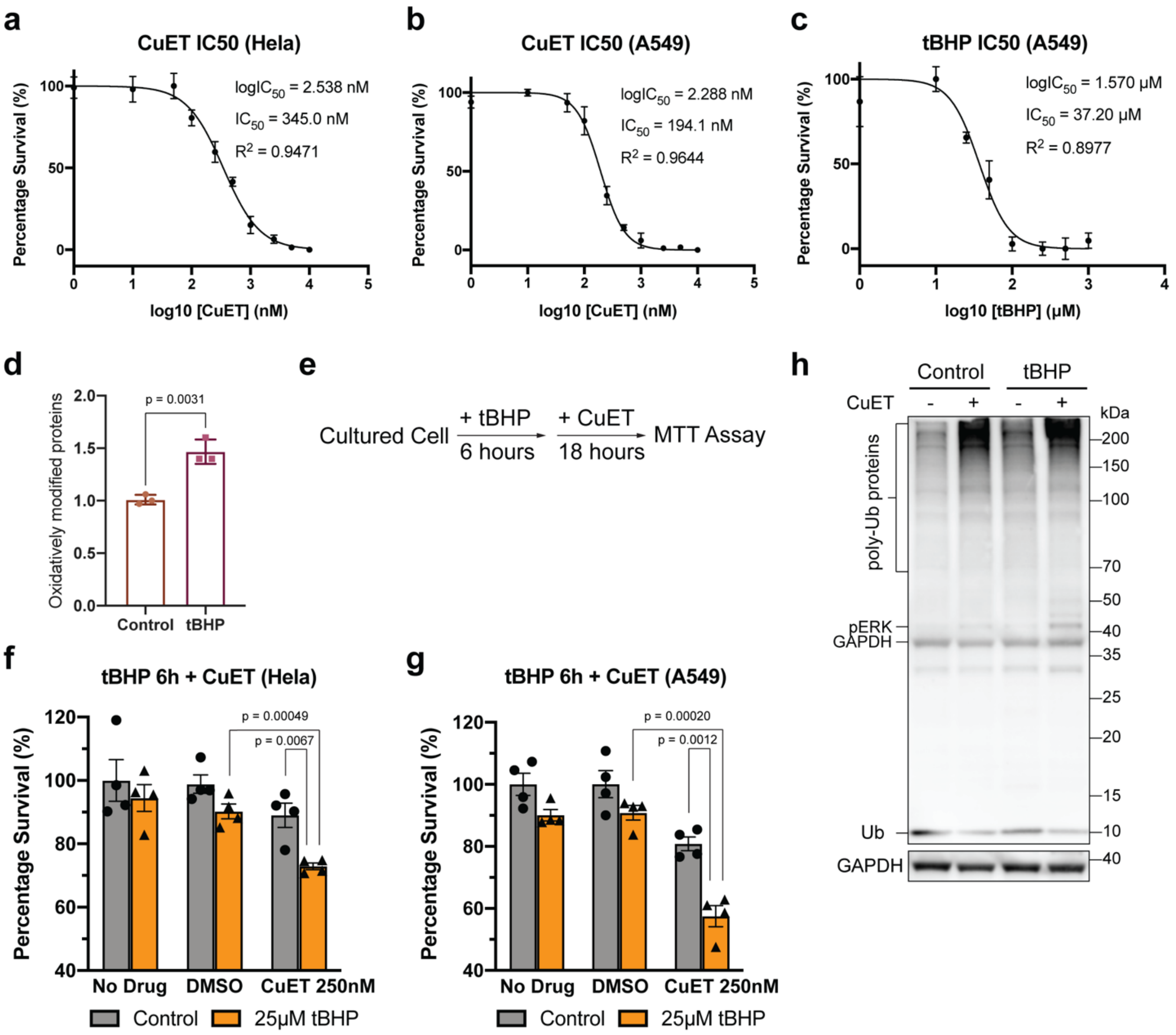

Supplementary Figure 8: The toxicity of CuET is enhanced by oxidative stress in cultured cancer cells.

(**a, b**) The dose-response curves of Hela (**a**) and A549 (**b**) cells treated with CuET. Cell viability under various doses of CuET was measured by MTT assay after 48 hours. The CuET concentration at which cell viability reaches 50% (IC50 values) was determined by non-linear fit in Prism (GraphPad) (n = 4 biological replicates, mean ± SEM). (**c**) The dose-response curve of A549 cells treated with oxidizing agent tert-Butyl hydroperoxide (tBHP). Cell viability was measured after 1 day (n = 3 biological replicates, mean ± SEM). (**d**) tBHP treatment induced the level of oxidatively modified proteins in A549 cells. The level of oxidatively modified proteins were measured using the Oxyblot Protein Oxidation Detection Kit (Millipore, S7150) (n = 3 biological replicates, mean ± SD, two-sided Student's t-test). (**e-g**) Pre-treatment of tBHP to

introduce oxidative state in the cultured cancer cells significantly enhanced the toxicity of CuET. (**e**) Experimental scheme of evaluating CuET in oxidative conditions. Hela (**f**) and A549 (**g**) cells were treated with 25 μM tBHP for 6 hours before treated with 250nM CuET for 18 hours. Cell viability was measured by MTT assay (n = 4 biological replicates, mean ± SEM, two-sided Student's t-test). (**h**) Cellular ubiquitination level of A549 cells after the pretreatment with and without 2.5 mM tBHP, and the treatment with and without 5 μM CuET, respectively. A Western blot of A549 cell lysate using anti-ubiquitin antibody (Abcam, ab19247, 1:3000) is shown. The experiment was repeated 3 times with similar results.

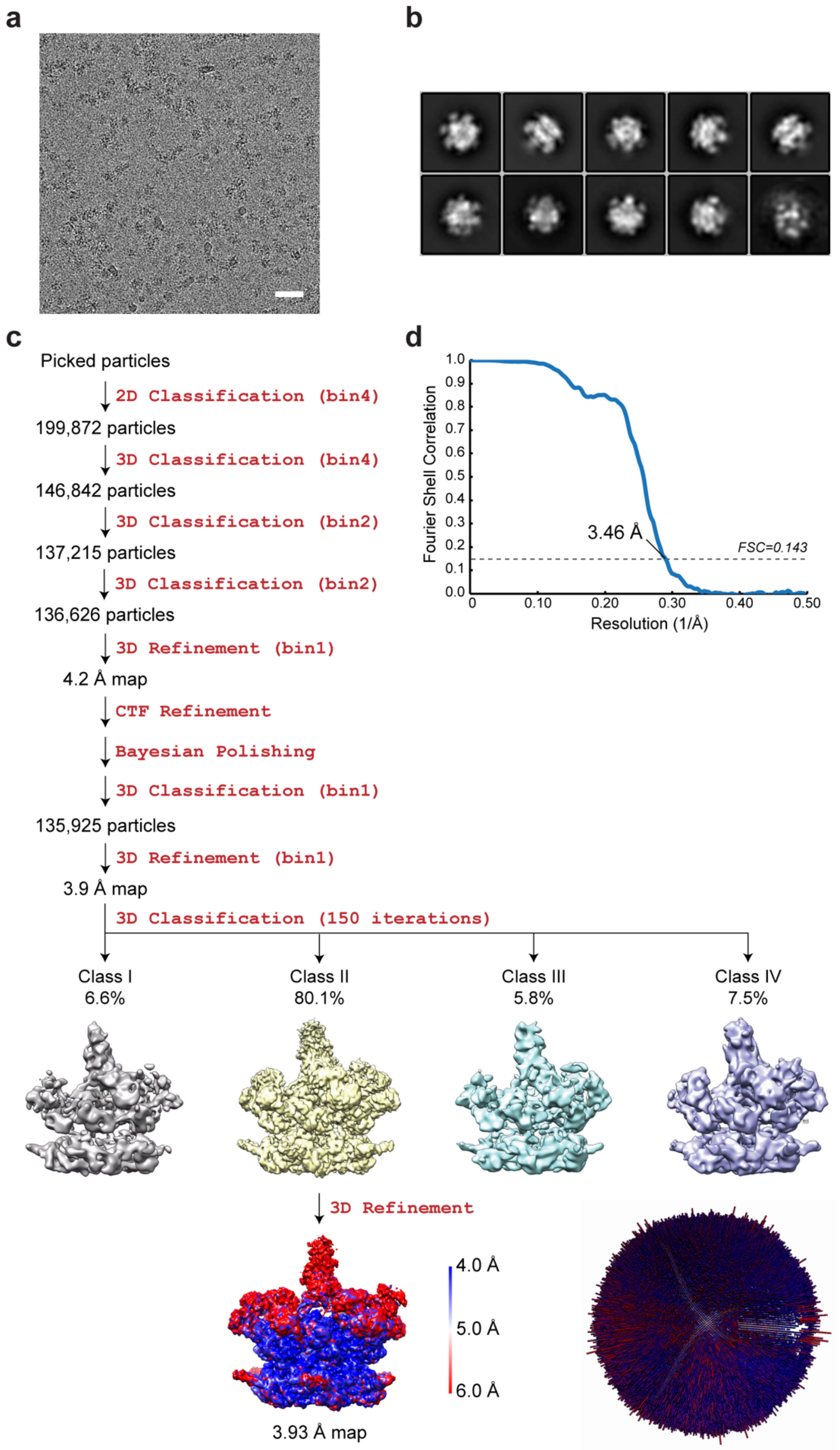
a
b
c
Picked particles
2D Classification (bin4)
199,872 particles
3D Classification (bin4)
146,842 particles
3D Classification (bin2)
137,215 particles
3D Classification (bin2)
136,626 particles
3D Refinement (bin1)
4.2 Å map
CTF Refinement
Bayesian Polishing
3D Classification (bin1)
135,925 particles
3D Refinement (bin1)
3.9 Å map
3D Classification (150 iterations)
Class I
6.6%
Class II
80.1%
Class III
5.8%
Class IV
7.5%
3D Refinement
4.0 Å
5.0 Å
6.0 Å
3.93 Å map
d
Fourier Shell Correlation
3.46 Å
FSC=0.143
Resolution (1/Å)
1.0
0.9
0.8
0.7
0.6
0.5
0.4
0.3
0.2
0.1
0.0
0
0.10
0.20
0.30
0.40
0.50

Supplementary Figure 9: Single-particle cryo-EM analyses for the complex of p97 and Npl4/Ufd1 in the presence of cupric ion.

(**a**) A representative micrograph. The scale bar corresponds to 50 nm. (**b**) Representative 2D class averages. The box edge corresponds to 290 Å. (**c**) The workflow of data processing. The dataset was subjected to particle selection, 2D classification, and multiple rounds of 3D classification. The maps after the last round of 3D classification were shown. All four maps are close to State II of the complex in the absence of cupric ion. The local resolution of the refined map was calculated using LocalRes[2]. The resolution after Relion 3D refinement and before post processing was listed. The distribution of the Euler angle (half sphere) corresponding to the local resolution map was shown. (**d**) Fourier shell correlation curves of the masked map after Relion post processing. The resolution was determined by the FSC=0.143 criterion.

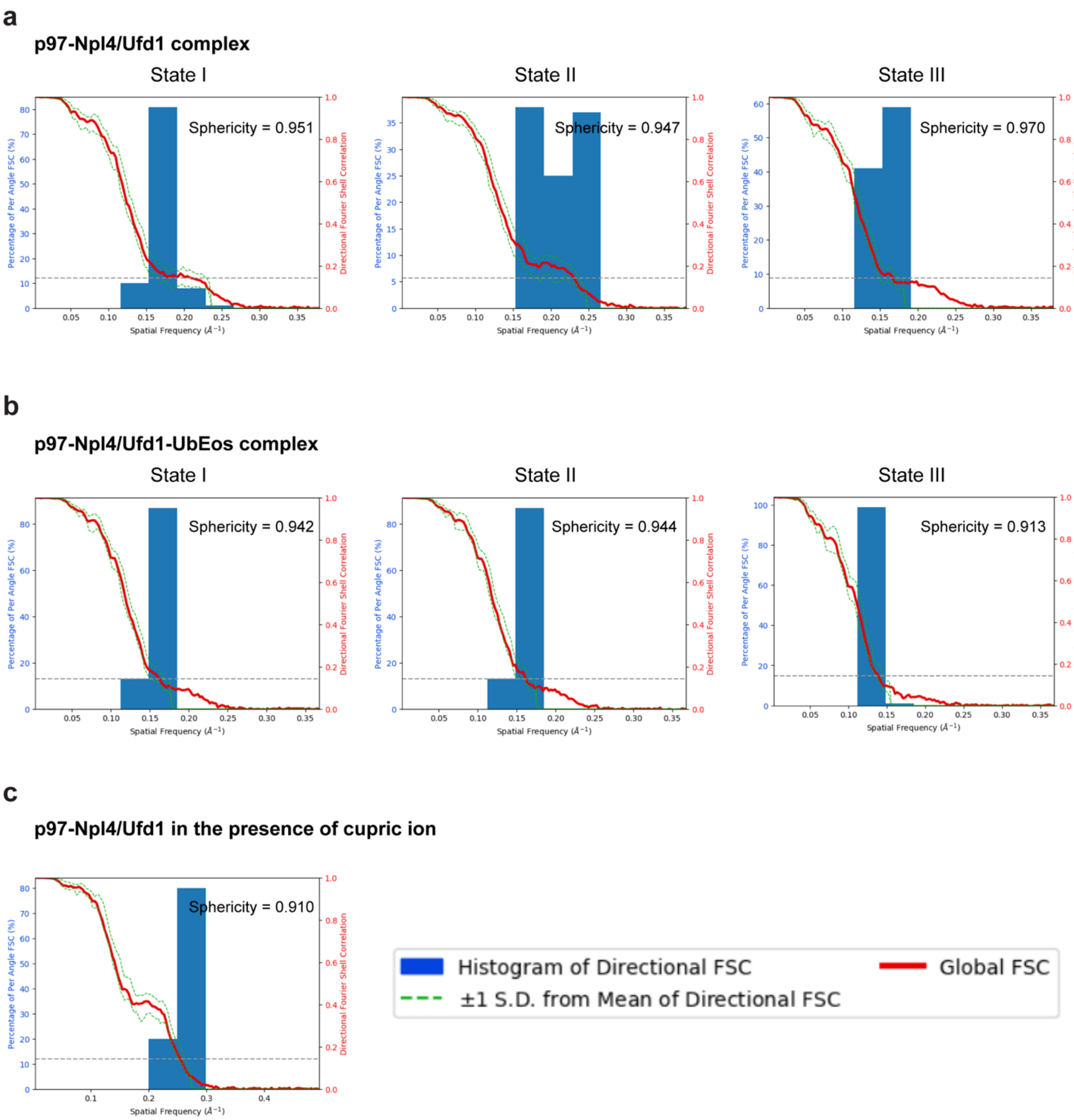

Supplementary Figure 10: Histograms and directional FSC plots for the cryoEM structures determined in this study.

(**a**) p97-Npl4/Ufd1 complex (corresponding to **Supplementary Fig. 1**). (**b**) p97-Npl4/Ufd1-UbEos complex (corresponding to **Supplementary Fig. 3**). (**c**) p97-Npl4/Ufd1 complex in the presence of cupric ion (corresponding to **Supplementary Fig. 9**). The plots were generated using the online 3DFSC server (https://3dfsc.salk.edu/upload/)[5].

Supplementary Table 1: Statistics of cryo-EM data collection and processing

	p97-Npl4/Ufd1				p97-Npl4/Ufd1-UbEos			p97-Npl4/Ufd1-Cu^{2+}
Microscope	Krios (NCI)				Krios (NCI)			Krios (UMich)
Magnification	105,000				64,000			81,000
Voltage (kV)	300				300			300
Spherical aberration (mm)	2.7				2.7			2.7
Detector	K2-Summit				K3			K2-Summit
Camera mode	Super resolution counting				Super resolution counting			Counting
Exposure rate (e^-/pixel/s)	7.25				24.3			6.4
Total exposure (e^-/Å^2)	50				50			50
Defocus range (μm)	-1.0 to -2.5				-1.0 to -2.5			-1.0 to -2.5
Pixel size (Å)	0.66 (1.32 physical)				0.68 (1.36 physical)			1.01
Mode of data collection	Stage position				Image shift			Stage position
Energy filter	20 eV slit				20 eV slit			No
Software for data collection	Latitude S				Latitude S			Leginon
Number of micrographs	1,920				7,490			2,974
Box size (pixel)	288				288			288
Initial particle images (no.)	576,987				1,056,857			243,163
Particle images for 3D (no.)	370,663				904,876			199,872
Symmetry imposed	C1			C6	C1			C1
	State I	State II	State III	p97 alone (masked)	State I	State II	State III	-
Final particle images (no.)	56,631	44,856	31,119	175,755	109,001	112,932	56,528	108,876
Map resolution, unmasked (Å)	6.0	4.6	4.3	-	6.2	7.1	7.3	3.9
Map resolution, masked (Å)	3.8	3.7	3.9	2.9	4.2	4.3	4.5	3.5
B-factor estimated (Å^2)	68.7	59.2	63.3	71.1	137	139	131	69.8
EMD accession code	21824	21825	21826	22521	21827	21828	21829	21830

Supplementary Table 2: Statistics of cryo-EM model refinement and geometry (p97 part)

**Model**		
Composition (#)		
Chains	8	
Atoms	34728 (Hydrogens: 0)	
Residues	Protein: 4386 Nucleotide: 0	
Water	0	
Ligands	MG: 12	
	AGS: 12	
Bonds (RMSD)		
Length (Å) (# > 4σ)	0.009 (0)	
Angles (°) (# > 4σ)	0.661 (3)	
MolProbity score	1.77	
Clash score	9.68	
Ramachandran plot (%)		
Outliers	0.00	
Allowed	3.85	
Favored	96.15	
Rotamer outliers (%)	0.00	
Cβ outliers (%)	0.00	
Peptide plane (%)		
Cis proline/general	7.7/0.0	
Twisted proline/general	0.0/0.0	
CaBLAM outliers (%)	0.98	
ADP (B-factors)		
Iso/Aniso (#)	34728/0	
min/max/mean		
Protein	5.10/91.30/41.80	
Ligand	18.75/33.80/33.43	
**Data**		
Box		
Lengths (Å)	175.56, 170.28, 114.84	
Angles (°)	90.00, 90.00, 90.00	
Supplied Resolution (Å)	2.8	
Resolution Estimates (Å)	Masked	Unmasked
d model	3.0	3.0
d FSC model (0/0.143/0.5)	2.8/2.8/2.9	2.9/2.9/3.0
Map min/max/mean	-0.18/0.41/0.00	
**Model vs. Data**		
CC (mask)	0.82	
CC (box)	0.74	
CC (peaks)	0.72	
CC (volume)	0.79	
Mean CC for ligands	0.83	

Supplementary Table 3: Primers used in this study

**Primer information**	
Name	Sequence
pET-47(b)-VCP-f	ggacccgggtaccagatggcctctggagccga
pET-47(b)-VCP-r	ggcctgtacagaattcgttagccatacaggtcatcgtcattgtct
pET-47(b)-VCP-A232E-f	gagattggtgtaaagcctcctcgg
pET-47(b)-VCP-A232E-r	ctttacaccaatctccttgaagagcgcaggatg
pET-47(b)-VCP-E578Q-f	cagttagattcaattgccaaggctcg
pET-47(b)-VCP-E578Q-r	caattgaatctaactgatcaaagaagagtacac
pET-47(b)-VCP-R113A-f	gctatccacgttctacccatcg
pET-47(b)-VCP-R113A-r	gtagaacgtggatagctttgccatactttacatc
NPL4-ZF1_Mutation-f	ggtcccttgggcaaggccgtggctgctgtcccactggagcc
NPL4-ZF1_Mutation-r	cttgcccaagggaccagcgcgagctaactgtgggtctcggctcc
NPL4-ZF2_Mutation-f	ctcccttggccaaatggtattgccaccaaagctcagcctagtgccatcac
NPL4-ZF2_Mutation-r	aataccatttggccaagggagggctccctcggctcctgacttaatc

Supplementary References

1. Pettersen, E. F. *et al.* UCSF Chimera--a visualization system for exploratory research and analysis. *J. Comput. Chem.* **25**, 1605–12 (2004).
2. Kucukelbir, A., Sigworth, F. J. & Tagare, H. D. Quantifying the local resolution of cryo-EM density maps. *Nat. Methods* **11**, 63–5 (2014).
3. Skrott, Z. *et al.* Alcohol-abuse drug disulfiram targets cancer via p97 segregase adaptor NPL4. *Nature* **552**, 194 (2017).
4. Waterhouse, A. *et al.* SWISS-MODEL: Homology modelling of protein structures and complexes. *Nucleic Acids Res.* **46**, W296–W303 (2018).
5. Tan, Y. Z. *et al.* Addressing preferred specimen orientation in single-particle cryo-EM through tilting. *Nat. Methods* **14**, 793–796 (2017).
