# Supplementary material for: Seesaw conformations of Npl4 in the human p97 complex and the inhibitory mechanism of a disulfiram derivative
Source: Nat Commun. 2021 Jan 5;12:121. doi: 10.1038/s41467-020-20359-x (PMC7785736; doi:10.1038/s41467-020-20359-x)
Supplement: Supplementary file 3 — Reporting Summary [file 41467_2020_20359_MOESM3_ESM.pdf]

natureresearch

Corresponding author(s): Minglei Zhao, Lei Liu

Last updated by author(s): Nov 15, 2020

Reporting Summary

Nature Research wishes to improve the reproducibility of the work that we publish. This form provides structure for consistency and transparency in reporting. For further information on Nature Research policies, see Authors & Referees and the Editorial Policy Checklist .

Statistics

For all statistical analyses, confirm that the following items are present in the figure legend, table legend, main text, or Methods section.

n/a	Confirmed	
☐	☒	The exact sample size (*n*) for each experimental group/condition, given as a discrete number and unit of measurement
☐	☒	A statement on whether measurements were taken from distinct samples or whether the same sample was measured repeatedly
☐	☒	The statistical test(s) used AND whether they are one- or two-sided<br>*Only common tests should be described solely by name; describe more complex techniques in the Methods section.*
☒	☐	A description of all covariates tested
☒	☐	A description of any assumptions or corrections, such as tests of normality and adjustment for multiple comparisons
☐	☒	A full description of the statistical parameters including central tendency (e.g. means) or other basic estimates (e.g. regression coefficient) AND variation (e.g. standard deviation) or associated estimates of uncertainty (e.g. confidence intervals)
☐	☒	For null hypothesis testing, the test statistic (e.g. *F*, *t*, *r*) with confidence intervals, effect sizes, degrees of freedom and *P* value noted<br>*Give P values as exact values whenever suitable.*
☒	☐	For Bayesian analysis, information on the choice of priors and Markov chain Monte Carlo settings
☒	☐	For hierarchical and complex designs, identification of the appropriate level for tests and full reporting of outcomes
☒	☐	Estimates of effect sizes (e.g. Cohen's *d*, Pearson's *r*), indicating how they were calculated

Our web collection on statistics for biologists contains articles on many of the points above.

Software and code

Policy information about availability of computer code

Data collection	Latitude S
Data analysis	MotionCor2 1.3.1, Relion 3.0.8, Ctffind4 4.1.10, ResMap 1.95, PyMol 2.2.3, Phenix 1.17.1, Chimera 1.13, ChimeraX 1.0, GraphPad Prism 8.2.1, ImageJ 2.0.0 (Fiji), OriginPro 2019b, Coot 0.9.1, CIMAGE

For manuscripts utilizing custom algorithms or software that are central to the research but not yet described in published literature, software must be made available to editors/reviewers. We strongly encourage code deposition in a community repository (e.g. GitHub). See the Nature Research guidelines for submitting code & software for further information.

Data

Policy information about availability of data

All manuscripts must include a data availability statement. This statement should provide the following information, where applicable:
- Accession codes, unique identifiers, or web links for publicly available datasets
- A list of figures that have associated raw data
- A description of any restrictions on data availability

cryo-EM maps have been deposited in the Electron Microscopy Data Bank (EMDB) under the accession codes EMD-21824 [https://www.emdataresource.org/EMD-21824] (p97-Npl4/Ufd1, State I), EMD-21825 [https://www.emdataresource.org/EMD-21825] (p97-Npl4/Ufd1, State II), EMD-21826 [https://www.emdataresource.org/EMD-21826] (p97-Npl4/Ufd1, State III), EMD-21827 [https://www.emdataresource.org/EMD-21827] (p97-Npl4/Ufd1-UbEos, State I), EMD-21828 [https://www.emdataresource.org/EMD-21828] (p97-Npl4/Ufd1-UbEos, State II), EMD-21829 [https://www.emdataresource.org/EMD-21829] (p97-Npl4/Ufd1-UbEos, State III), EMD-21830 [https://www.emdataresource.org/EMD-21830] (p97-Npl4/Ufd1 in the presence of cupric ion), and EMD-22521 [https://www.emdataresource.org/EMD-22521] (p97-Npl4/Ufd1, masked around p97 and applied C6 symmetry). The atomic model of p97 bound to ATPγS has been deposited in the Protein Data Bank (PDB) under the accession code 7JY5 [https://www.rcsb.org/structure/unreleased/7JY5]. Source data are provided with this paper.

Field-specific reporting

Please select the one below that is the best fit for your research. If you are not sure, read the appropriate sections before making your selection.

☒ Life sciences ☐ Behavioural & social sciences ☐ Ecological, evolutionary & environmental sciences

For a reference copy of the document with all sections, see nature.com/documents/nr-reporting-summary-flat.pdf

Life sciences study design

All studies must disclose on these points even when the disclosure is negative.

Sample size	All experiments involving tissue culture were performed in three or four independent culture preparations.
Data exclusions	No data was excluded.
Replication	Three or four independent biological replications were performed for the cell viability assays, quantification of intracellular copper level, and quantification of oxidatively modified proteins after tBHP treatment. Three technical replicates were performed for the substrate unfolding assay to estimate reading errors of fluorescent signals.
Randomization	Randomization is not relevant to this study.
Blinding	Blinding is not relevant to this study.

Reporting for specific materials, systems and methods

We require information from authors about some types of materials, experimental systems and methods used in many studies. Here, indicate whether each material, system or method listed is relevant to your study. If you are not sure if a list item applies to your research, read the appropriate section before selecting a response.

Materials & experimental systems

n/a	Involved in the study	
☒	☐	Antibodies
☐	☒	Eukaryotic cell lines
☒	☐	Palaeontology
☒	☐	Animals and other organisms
☒	☐	Human research participants
☒	☐	Clinical data

Methods

n/a	Involved in the study	
☒	☐	ChIP-seq
☒	☐	Flow cytometry
☒	☐	MRI-based neuroimaging

Eukaryotic cell lines

Policy information about cell lines

Cell line source(s)	Hela cells (ATCC CCL-2) were kindly provided by Dr. Chuan He at University of Chicago and originally from ATCC.<br>A549 cells (ATCC CCL-185) were a gift from Dr. Eileen Dolan at University of Chicago and originally from ATCC.
Authentication	None of the cell lines used were authenticated.
Mycoplasma contamination	Mycoplasma were tested by PCR-based method and no mycoplasma contamination were detected in all cell lines used.
Commonly misidentified lines (See ICLAC register)	No commonly misidentified lines were used in the study.
